# The Primary Prevention of PTSD in Firefighters: Preliminary Results of an RCT with 12-Month Follow-Up

**DOI:** 10.1371/journal.pone.0155873

**Published:** 2016-07-06

**Authors:** Petra M. Skeffington, Clare S. Rees, Trevor G. Mazzucchelli, Robert T. Kane

**Affiliations:** School of Psychology and Speech Pathology, Faculty of Health Sciences, Curtin University, Perth, Australia; University of Pennsylvania, UNITED STATES

## Abstract

**Aim:**

To develop and evaluate an evidence-based and theory driven program for the primary prevention of Post-traumatic Stress Disorder (PTSD).

**Design:**

A pre-intervention / post-intervention / follow up control group design with clustered random allocation of participants to groups was used. The “control” group received “Training as Usual” (TAU).

**Method:**

Participants were 45 career recruits within the recruit school at the Department of Fire and Emergency Services (DFES) in Western Australia. The intervention group received a four-hour resilience training intervention (Mental Agility and Psychological Strength training) as part of their recruit training school curriculum. Data was collected at baseline and at 6- and 12-months post intervention.

**Results:**

We found no evidence that the intervention was effective in the primary prevention of mental health issues, nor did we find any significant impact of MAPS training on social support or coping strategies. A significant difference across conditions in trauma knowledge is indicative of some impact of the MAPS program.

**Conclusion:**

While the key hypotheses were not supported, this study is the first randomised control trial investigating the primary prevention of PTSD. Practical barriers around the implementation of this program, including constraints within the recruit school, may inform the design and implementation of similar programs in the future.

**Trial Registration:**

Australian New Zealand Clinical Trials Registry (ANZCTR) ACTRN12615001362583

## Introduction

Exposure to traumatic events is a known risk factor for post-traumatic stress disorder (PTSD), fire and emergency service workers have increased exposure to these events and therefore represent an at-risk group for PTSD as well as depression, anxiety, sleep difficulties, problematic alcohol use, relationship breakdown, and suicide [1–3]. The estimated prevalence of PTSD in fire and emergency service workers is 17%-22%, compared to a lifetime prevalence of 1%-8% in the general population [3]. It is critical to fire and emergency services and other high risk professions that efforts are made to develop a comprehensive plan for managing psychological stressors [4], and there are increasing directives that government agencies should be made personally responsible for the psychological health (as a result of potentially traumatic event [PTE] exposure) of their staff, that active efforts to boost resilience to trauma be made, and that cultural changes be prompted to reduce stigma and diminish barriers to treatment seeking [5]. However, studies utilising robust methodology to test the effectiveness of PTSD primary prevention programs are rare [6]. Some programs have been criticised for their lack of scientific rigour prior to application, such as the universal implementation of the Comprehensive Soldier Fitness program with the U.S. Army [7]. The aim of the present study was to conduct a scientifically rigorous evaluation of a theory driven primary prevention program with trainee fire fighters in Western Australia.

Surprisingly, less research has been conducted around the prevention of PTSD as opposed to secondary interventions or treatments. The vast majority of research on PTSD prevention, consisting predominantly of tests of psychological debriefing, have shown relatively little efficacy in preventing PTSD [8], and there is little research on factors other than post-trauma social support that might alter either perceptions of threat or the development of PTSD [9]. Current intervention strategies include secondary or early intervention (aimed at those at high risk due to recent PTE exposure; [10] and tertiary intervention in the form of Acute Stress Disorder treatments (aimed at those who have current symptoms of pathology [11]. There is little research on the primary prevention of PTSD [6]. The prevention of PTSD would be valuable in sparing individuals, families and organisations the financial, personal and social costs of this serious disorder [12–14].

### The Mental Agility and Psychological Strength Training Program

The Mental Agility and Psychological Strength (MAPS) training program is a universal intervention for the primary prevention of PTSD that was developed from the ground up following a systematic review of PTSD prevention literature [6] and consultation with PTSD experts and key fire and emergency services stakeholders in Western Australia. The MAPS program focusses on building knowledge of psychological wellbeing and PTSD as well as practical skills such as cognitive re-structuring, support seeking, and self-soothing or self-moderating, all of which are factors in the aetiology of PTSD and other post-trauma pathologies [15].

A key risk factor for PTSD is maladaptive cognitive appraisal. Unhelpful appraisals of stress responses and trauma reactions, as well as isolation and avoidance behaviours fit within a cognitive model of PTSD as precipitating and perpetuating factors of the disorder [16–18]. It can be argued that these factors also contribute to common comorbid issues, such as drug and alcohol use, relationship breakdown, depression, and additional anxiety diagnoses [9,19,20]. Fire fighters’ reports indicate that a sense of helplessness over a traumatic situation was often critical in terms of their emotional response; many have reported events in which their physical safety was not endangered but they felt threatened by their inability to manage the physical or emotional trauma being suffered by the victim [21]. Given the information we have to-date about the importance of appraisal in the development of PTSD, the MAPS program includes cognitive and psychoeducational components that may facilitate helpful and adaptive appraisals of stressful or potentially traumatic situations.

Social support, camaraderie and support seeking are frequently cited factors that buffer individuals against both psychological and physical disease [22]. Durham and McCammon [23] reported that rescue workers appeared to infrequently seek out emotional support, with only 11% of their sample agreeing that they sought emotional support from others. This would suggest that there exists a need for educating fire fighters in the importance of using a range of support systems, including emotional support [24]. It is frequently reported that fire fighters tend to repudiate any notions toward help-seeking behaviours, and so the MAPS program includes an emphasis on identifying and utilising social supports to maintain mental health.

Peri-traumatic arousal and a sense of being out of control of personal reactions during a PTE have been correlated with PTSD development [16,25]. It has been suggested that mindfulness skills may improve the regulation of emotions associated with traumatic and other stressful events [26]. Past research has found promising results in boosting psychological resilience with the provision of relaxation and mindfulness based training prior to exposure to trauma [6,27–29] and so mindfulness and relaxation training was also included as part of the MAPS program.

### Summary

For some time it has been stated by government bodies, researchers and academics that there is a clear need for more research into the primary prevention of PTSD within high risk professions [5,6,24], although no robust research has been published in this area to-date despite the clear need to apply research to interventions that translate into the real world [30]. Through systematic critical analysis of the literature [6] and consultation with clinicians and key stakeholders, the MAPS program was developed as an evidence based and theory driven program aimed at the primary prevention of PTSD in early career fire fighters at the Department of Fire and Emergency Services in Western Australia. The aim of the current study was to conduct a rigorous evaluation of the MAPS program in a randomised control trial with a 12-month follow up.

### Hypotheses

It was anticipated that, compared to the Training-As-Usual (TAU) group, the intervention group would show a significant increase in trauma knowledge and significant improvements in perceived social support and coping from pre-test to 6- and 12-month follow-ups. Also, compared to the intervention group, the TAU group would show a significantly greater increase in pathology from pre-test to 6-month and 12-month follow-ups.

## Method

### Participants

Participants were 73 male and 4 female Trainee Firefighters (TFFs) at the DFES Training Academy in Perth, WA. Trainees underwent psychological screening prior to acceptance into recruit school (the details of this psychological screening are not available) and did not meet diagnostic criteria for any mental health conditions at baseline. All trainees commencing training during the duration of the data collection period (June 2013- December 2014) were invited to participate. Participation was voluntary and all participants gave informed consent. There were no exclusion criteria. All participants were invited to participate in the study during their first week of recruit school, at which time they completed baseline measures if consent was given. For futher information regarding ethical considerations in relation to this study please see Supporting Information files S1 Document. Ethics Form A and S2 Document. Ethics Form B.

### Measures

#### Demographics

A short demographic questionnaire to gather information relating to age and gender.

#### MAPS knowledge

A 20-item multiple choice assessment of trauma knowledge, developed specifically for this study, to assess whether the psycho-educational component of the intervention improves knowledge of trauma. Items on this assessment were directly related to information provided during the psycho-educational phase of the intervention (see Appendix A).

#### PTE exposure

The Traumatic Stress Schedule [31] is a 9-item instrument developed to examine lifetime exposure to nine types of potentially traumatic events and has been shown to have good stability, test-retest validity and symptom reliability (Norris & Hamblen, 2004). The TSS was used in this study to measure lifetime exposure to potentially traumatic events that may have been encountered in both private and professional experiences. This is an objective measure of PTE exposure and does not account for the subjective experience of any given PTE.

#### PTSD symptoms

The PTSD Checklist—Civilian Version (PCL-C;[32]) was used to assess PTSD symptom presence and severity. The PCL-C is a 17-item inventory that assesses the specific symptoms of PTSD; that has demonstrated internal consistency, test-retest reliability, convergent validity and discriminant validity [33]. Cronbach’s alpha coefficients indicate high internal consistency [33]. The respondent is asked to rate how much the problem described in each statement has bothered him or her over the past month on a 5-point scale ranging from 1 (not at all) to 5 (extremely). A total score is an indicator of PTSD symptom severity [34]. Scores on the PCL-C may be reported as a total, indicating PTSD symptom severity with an overall cut-off warranting further assessment. As outlined by Blanchard and colleagues [32] the diagnostic efficiency of the PCL-C can be improved by individually interpreting item scores and assessing positive endorsement of each symptom cluster rather than a total score. Items are considered to endorse a symptom if they are rated at 3 or higher. This scoring method more accurately reflects the DSM-IV diagnostic criteria. By extending the methodology for deriving a DSM-IV diagnosis from PCL responses, an estimate using the DSM-5 criteria can also be calculated.

#### Other mental health symptoms

A short version of the DASS [35] was administered to measure any current symptoms of depression, anxiety, and stress. The DASS is a set of three self-report scales designed to measure the negative emotional states of depression, anxiety, and stress. Each of the three DASS scales contains 14 items, divided into subscales of 2–5 items with similar content. Subjects are asked to use 4-point severity/frequency scales to rate the extent to which they have experienced each state *over the past week*. Scores for Depression, Anxiety, and Stress are calculated by summing the scores for the relevant items. The DASS-21 has been shown to distinguish well between features of depression, physical arousal, and psychological tension and agitation, and the internal consistency and concurrent validity of this measure are in the acceptable to excellent ranges [36].

#### Perceived social support

The Social Support Questionnaire—Short Form [37] is used to quantify the availability and satisfaction with social support. It is a 27-item self-administered scale that has demonstrated high internal consistency reliability and test-retest reliability. A support score for each item is calculated by the number of individuals the participant listed (number score). The overall support score (SSQN) is calculated by the mean of this score across the items. The overall satisfaction score is calculated by taking the mean of the satisfaction scores.

#### Coping strategies

The Brief Coping Orientations to Problems Experienced (Brief COPE) scale is a 28-item scale used to measure a broad range of cognitive and behavioural coping strategies that individuals typically use in stressful situations [38]. It includes 14 subscales: active coping, planning, positive reframing, acceptance, humour, religion, emotional support, instrumental support, self-distraction, denial, venting, substance use, behavioural disengagement, and self-blame. Cronbach’s alpha-coefficients ranged between .74 and .96. The Brief COPE has been shown to be psychometrically similar to the full COPE inventory, with acceptable test-retest and internal consistency reliability, as well as acceptable external validity (Carver, 1997).

The authors recommend that the Brief COPE be interpreted as the 14 aforementioned subscales, however there is evidence that the scale may also be divided into “adaptive” or “maladaptive” subscales. The adaptive subscale includes the active coping, planning, positive reframing, acceptance, humour, religion, emotional support, and instrumental support subscales, with possible scores ranging from 0 to 48. The maladaptive subscale comprises the self-distraction, denial, venting, substance use, behavioural disengagement, and self-blame subscales, with possible scores ranging from 0 to 36. These subscales have been similarly categorised as adaptive and maladaptive in other mental health and stress research [39–41].

### Study Design and Procedure

An experimental design was used to evaluate the program. In the present study, MAPS was delivered in a selective fashion; individuals at risk of PTSD due to their profession were targeted. A pre-intervention / post-intervention / follow-up TAU group design with clustered random allocation of participants to groups was used. TFFs within DFES are naturally grouped into “schools”, where a school is a cohort that completes training together. For this reason, random allocation of single subjects to treatment or TAU groups is not feasible. Rather, schools were randomly allocated to treatment or TAU. Random allocation was decided by entering the name of each condition into sealed envelopes; an envelope was drawn by the first author to determine condition at the commencement of each participating recruit school.

The TAU group was treated identically to the intervention group, proceeding through all components of DFES recruit training, but did not participate in the intervention program. Due to the limited time and resources available within the DFES professional training program, an attention placebo TAU group was not a viable option. All participants were measured on the outcome variables during the first week of recruit training. All participants were measured once again on the outcome variables at 6 months post-graduation, and for one final time 12 months post-graduation.

Participants in the intervention group (*n* = 30; one school) received four group sessions of MAPS training in addition to the standard recruit school curriculum. Participants in the TAU groups (*n* = 45; two schools of 24 and 21 TFFs) completed the same measures at the same times as the intervention group. Participants were not compensated for their time, as the training comprised part of their professional training for which they were remunerated by DFES.

Following written consent, measures were administered pre-intervention (T1), at 6-month follow-up (T2) and at 12-month follow-up (T3). Data collection at T1 was done in person. At T2 and T3, an invitation to complete the measures online was delivered electronically to all participants. This research was approved by the Human Research Ethics Committee at Curtin University in October 2011 (HR113/2011) and was registered as a clinical trial with the Australian and New Zealand Clinical Trials Registry (ACTRN ACTRN12615001362583) after data collection was completed at the request of the publisher. The authors confirm that all ongoing and related trials for this intervention are registered.

### Intervention

The intervention program was delivered by the primary researcher on-site at DFES over four hours (four 1-hour sessions over four weeks). The researcher is a registered psychologist with a masters level qualification and experience in delivering psycho-education and training seminars and treating stress and trauma syndromes. Each one-hour MAPS session comprised a fully contained module. The MAPS précis was presented at the start and end of each session, as follows:

Creating strong MAPS:

1. Moment—Take a moment to choose the strongest option
2. Assess—Make an assessment of what the situation is, what is happening for you (internally and externally) and what outcome you would like.
3. Plan—Plan your course of action
4. Support—what support(s) might you need to follow through with the strongest possible response?

It should be noted that the program was initially written to be eight hours in length, as this was the shortest comparable resilience program length published at that time [42]. Due to timetable constraints within the recruit school this was not possible and so program length was reduced by half. It was also intended that the MAPS program be delivered by a suitably qualified and experienced independent DFES staff member, preferably a psychologist from the DFES Wellness Team. This was also not possible due to workload and time constraints within the DFES Wellness Team.

The MAPS program has a salutogenic focus on strength and draws parallels between mental and physical wellbeing, to normalise coping and efforts to maintain psychological wellbeing and promote a focus on mental health [43]. Research literature has indicated that often psycho-educational seminars and other similar programs for high-risk professions have a focus on trauma and negative trauma outcomes (such as PTSD); this was deliberately avoided in the MAPS program as it can give the impression that PTSD is the only outcome or a likely outcome following PTE exposure.

Module One was an introduction to the MAPS program. Participants were introduced to the presenter (Skeffington), given an overview of the MAPS program (as above) before being supported through group discussions and psychoeducational presentation slides about coping strategies, helpful versus unhelpful coping and planning coping strategies for mental strength. This included psycho-education about the expected physiological and neurobiological responses to stress. Information about PTSD was presented to facilitate correct identification of PTSD symptoms; this was presented as a possible, but not probable, outcome following trauma. Activities around coping strategies and identification and use of social supports were also included to encourage consideration of coping options and the final session provided a recap and information about ongoing self-care. Increasing knowledge about stress, PTE exposure and stress reactions, as well as broadening coping skills was intended to bolster self-efficacy and to encourage adaptive coping behaviours in response to stress. If threats and stress responses are perceived as more benign then the opportunity for self-regulation, resilience, and growth is improved [44].

The objective of Module Two was to instruct participants in how to “take a moment” to be able to choose their response while under stress. This included identifying features of mentally strong individuals, identifying thoughts and feelings and defusion tactics. The “ACT in a nutshell” and “thinking self versus observing self” activities [45] were used as experiential exercises to illustrate metacognitive concepts, such as how we identify and relate to thoughts and how we can “step back” or “defuse” from thoughts in order to choose our behaviour. These concepts were revisited and consolidated in Module Three, where the concept of identifying and using appropriate supports was added. The role of social support in mental and physical wellbeing was highlighted and participants identified their own emotional, instrumental and formal supports across personal and professional settings. Meaningful connections were also explored, including an activity based on identifying positive and negative connections and discussions of how to manage interpersonal stressors.

The final MAPS module targeted maintenance and self-care. It aimed to further normalise stress reactions, particularly in high-risk settings, and to improve self-awareness and indicators of stress. Participants completed worksheets that asked them to endorse symptoms of stress from their personal experience that may be used as early warning signs and individual self-care plans were completed. Group discussion around the importance of recognising stress and engaging in self-care as a way to maintain mental strength was facilitated.

The entirety of the MAPS program was facilitated in a Socratic style and an underlying theme of normalisation of stress reactions and reducing barriers to treatment or support seeking were present. For example, a recurring metaphor was drawn between physical and mental health. In terms of seeking support this metaphor was included:

*If I hurt my knee at the gym*, *I will know because of pain*, *restricted movement*, *and inability to continue exercising or bear weight on that leg*. *At first I can manage this myself—I can RICE (Rest*, *Ice*, *Compression*, *Elevation) and see if that helps*. *I might ask a friend or family member for physical assistance with things for a few days*. *If it is still painful after a few days (or over a week) of this*, *I would be thinking about getting a professional involved*. *I can go to the physio and if I go early*, *before more damage is caused*, *the physio can rehab me in a short amount of time*. *It is similar with my mental health*. *If I have a build-up of stress or one significantly stressful event*, *I can first think about managing this myself through rest and self-care*. *I might talk to friends and family and take it easy for a while*. *However*, *if after a few weeks it is still bothering me*, *it might be time to get a professional involved*. *I can talk to the Wellness Team*, *use my EAP (Employee Assistance Program) or go to my GP for a referral to a private psychologist*. *As with my knee problem*, *the longer I leave it*, *the longer the rehab will be*.

During the introduction to MAPS and throughout the course a briefer version of this metaphor occurs, with the simple question “If I went to the gym once, two years ago, would I expect to be fit today?”, to reinforce the normality and ongoing nature of tending to self-care and mental health. Please see Table 1 for a full overview of the MAPS program.

**Table 1:** Overview of MAPS Content.

Module	Content	Supporting Evidence
**1**	**• Overview of MAPS**	**•** Sijaric Voloder, 2008
	**• How to create strong MAPS**	• Wolmer et al, 2011
	**• Introduce the physical fitness/mental fitness analogy**	**•** Sarason et al, 1979
	**• Extend the salutogenic analogy: mental fitness also requires ongoing practice, sometimes will need professional assistance and both mental and physical wellness should be attended to for overall fitness and wellbeing**	
	**• Helpful vs. unhelpful coping strategies**	
	**• Choosing your response**	
**2**	**• Features of “mentally strong” people**	**•** Berceli & Napoli, 2006
	**• Taking a moment**	**•** Ozer et al, 2008
	**• Defusion exercise**	**•** Smith et al, 2011
	**• Benefits of daily practice**	
**3**	**• Seeking support—different types of support**	**•** Sarason et al, 1979
	**• Identifying appropriate supports**	**•** Armfield, 1994
	**• Identifying and using meaningful connections**	**•** Tuckey & Hayward, 2011
**4**	**• Self-care**	**•** Pearlman, 1995
	**• Identifying early signs of stress**	**•** Shapiro, Brown & Biegel, 2007
	**• Developing self-care strategies**	
	**• Final recap**	

### Power

According to the power program (G*Power 3.1), at a per-test alpha-level of .05, 82 participants (41 in each group) would be required for an 80% chance of capturing a “small” to “moderate” Group x Time interaction (*f* = .16). Using a repeated measures ANOVA estimate. The Generalised Linear Mixed Model (GLMM; the statistical procedure used to test the hypotheses, as outlined below) estimate would be approximately equivalent; but with GLMM, natural attrition can occur without seriously compromising power. This is because the GLMM maximum likelihood procedure is a full estimation procedure, which uses all of the data available at each time period without being dependent on each participant providing data at each time point [46].

### Treatment Adherence

All intervention sessions were observed by at least two members of the DFES Health and Wellness Team and senior officers involved with TFF instruction. The first author constructed a session checklist outlining the objectives of each intervention module. Prior to delivery of any intervention sessions, the checklist was examined by the second author to ensure content validity. Each impartial observer present for each intervention module (minimum of two observers for adherence ratings) was asked to rate adherence to module objectives on a 7-point Likert scale that ranges from (1) *not at all covered* to (7) *completely covered*. The inter-rater reliability of this measure was calculated and found to be strong (Pearson’s *r* = .91). Mean adherence to treatment protocol across sessions was rated as 6.77/7 (*SD* = 0.98).

### Hypothesis Testing

Each hypothesis predicts that the key dependent variables will change at a greater rate for the intervention group than the TAU group. These predictions are best tested with GLMM [46]. GLMM was used to analyse the outcome data within the context of a hierarchical design in which Time (T1, T2, and T3) was nested within participants, and participants were nested within schools, and schools were nested within group (intervention, TAU).

### Assumption Testing

The traditional ANOVA model for repeated measure designs assumes homogeneity of variance, normality, sphericity, and independence of observations [47]. The GLMM “robust statistics” option generally takes care of violations of normality and homogeneity of variance. Violations of sphericity can be accommodated by changing the covariance matrix from the default of compound symmetry to autoregressive. Finally, by specifying the multilevel nature of the current data (participant nested within schools) in the GLMM syntax, GLMM can accommodate intra-school dependencies in the outcome measures [48].

## Results

### Participant Flow

**Fig 1:**
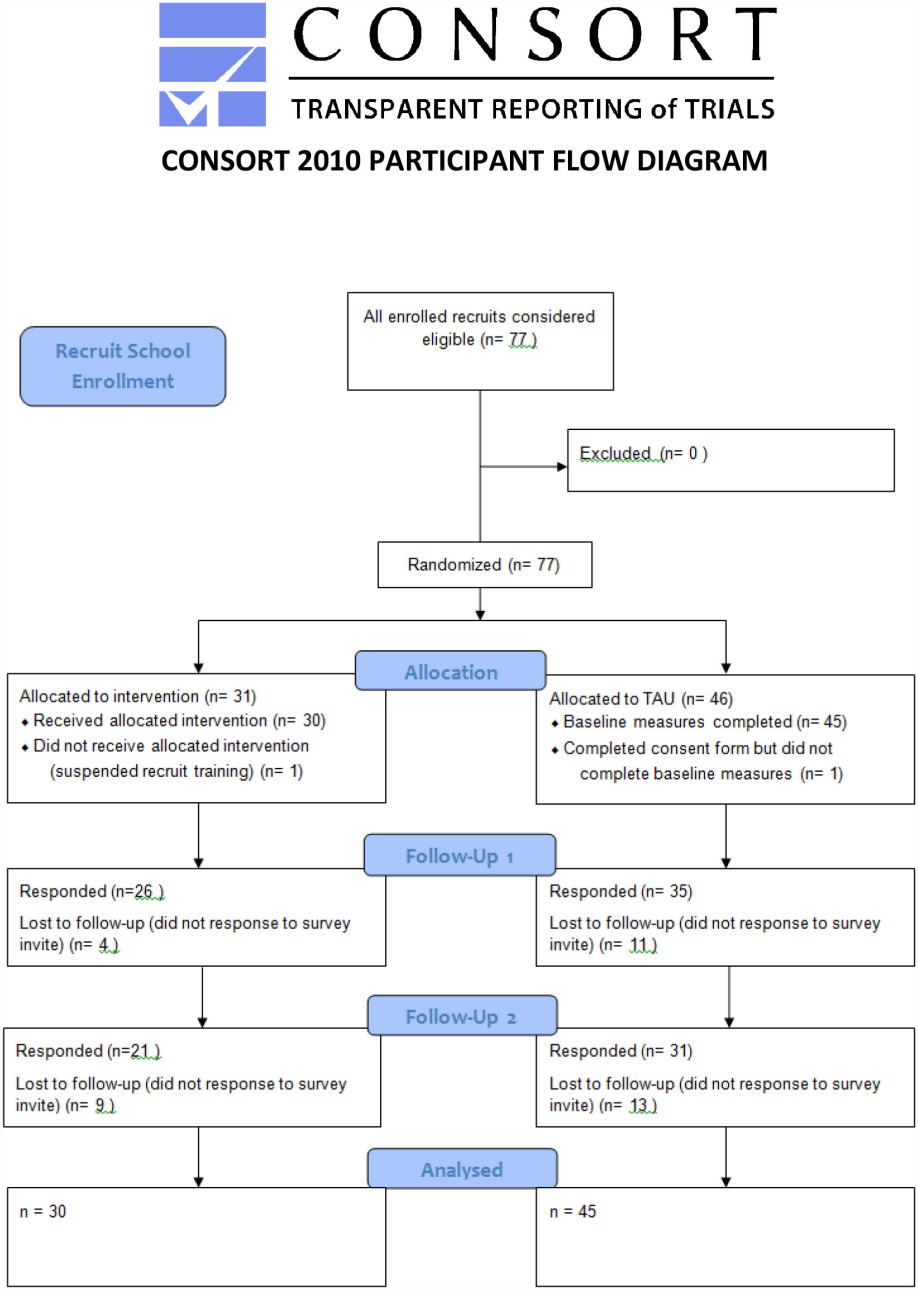
Participant Flow Diagram.

During the recruitment phase of this study, 77 participants within three separate TFF schools were recruited from June 2012 –December 2013, with 12 month follow up data collection occurring from June 2013-December 2014. One participant assigned to the TAU group signed the consent form but completed less than half of the baseline measures and was excluded from analyses. One participant assigned to the intervention group withdrew from TFF school temporarily due to personal circumstances and did not complete MAPS training or any subsequent measures and was thus excluded from analyses. Seventy-five participants remained. For a full outline of participant flow see Fig 1. The full CONSORT checklist can be found in Supporting File S3 Document. CONSORT Checklist.

### Tests of Sample Representativeness (External Validity)

The majority of participants were male, reflecting the gender bias in this high-risk profession [49]. A chi-square goodness-of-fit test indicated that gender was similarly distributed in the participants responding to the study as the DFES career firefighter population (χ^2^ [1] = .03, *p* = .866; OR = 1.36). A goodness-of-fit test for age was not run as it was not expected that members of the recruit school would reflect the age demographics of the organisation as a whole because recruit school members would be, on average younger and less experienced in fire and emergency work than career fire fighters. For all demographic details see Table 2. The complete data set for this study can be found in Supporting Information file S1 data. MAPS data.

**Table 2:** Demographic Characteristics (n. %) for Participants in the Intervention and TAU Conditions.

	Intervention (*n* = 30)	TAU (*n* = 45)	Two-sided *t*-test and Fisher’s exact test (only *p*-values are reported for the latter)	Entire sample (*N =* 75)	Effect Size
Mean age in years (*SD*)	29.23 (4.55)	28.58 (4.72)	*t*(73) = -.60, *p* = .552	28.85 (4.73)	*d* = -.141
Gender (male)	29 (96.7%)	44 (97.8%)	*p* = 1.00	73 (97.3%)	OR = .659
Marital Status					
Divorced	12 (40.0%)	22 (48.9%)	*p* = .738	34 (45.3%)	OR = .697
Never Married	17 (56.7%)	22 (48.9%)		39 (52.0%)	OR = 1.367
Married/De facto	1 (3.3%)	1 (2.2%)		2 (2.7%)	OR = 1.512

### Tests of Group Equivalence (Internal Validity)

Table 3 displays the baseline demographic and clinical characteristics for participants in the intervention and TAU conditions. Fisher’s exact tests (2-sided) indicated that participants in the intervention and TAU conditions did not significantly differ in terms of gender ratio or marital status. Independent samples *t*-tests indicated that participants in the intervention and TAU conditions did not differ significantly in age, PTSD symptoms, past trauma exposure, depressive symptoms, anxiety symptoms, stress symptoms, social support, adaptive coping and maladaptive coping at baseline (Table 3).

**Table 3:** Baseline Means, Standard Deviations and t-tests for Participants in the Intervention and TAU Conditions.

Measure	Intervention *M* (*SD*)	TAU *M* (*SD*)	*t-test t*(73)	*p*	Cohen’s d
Trauma Knowledge	16.60 (1.38)	16.64 (1.30)	0.14	.888	.032
PTSD symptoms	21.13 (5.16)	23.69 (7.54)	1.62	.110	.374
PTE exposure	2.27 (1.91)	1.73 (1.48)	-1.36	.179	-.314
Depression	0.53 (1.38)	1.56 (2.83)	1.84	.070	.425
Anxiety	2.93 (4.51)	2.76 (3.47)	-0.19	.848	-.044
Stress	5.40 (4.96)	5.11 (4.60)	-0.26	.797	-.060
Social Support	4.85 (1.90)	4.71 (2.16)	-0.29	.776	-.067
Adaptive Coping	37.60 (7.05)	36.89 (8.36)	-0.38	.702	-.088
Maladaptive Coping	18.23 (4.93)	17.93 (3.11)	-0.32	.747	-.074

### Trauma Exposure

At baseline 33 (73.3%) participants randomised to TAU and 24 (80.0%) randomised to intervention reported at least one prior PTE exposure. A chi-square test for goodness of fit (with α = .05) was used to assess whether there was a difference in previous PTE exposure across the intervention and TAU groups. Table 4 lists the percentages of TFFs endorsing each frequency of PTE exposure to date across each data collection time point. The Chi-square test at baseline was not statistically significant, χ^2^ (6, *N* = 75) = 10.60, *p* = .101. At Time 2 80.4% of the TAU group and 80.0% of the intervention group reported some form of lifetime PTE exposure, χ^2^ (6, *N* = 61) = 5.49, *p* = .483. At Time 3 90.9% of the TAU group and 90.0% of the intervention group reported some form of lifetime PTE exposure, χ^2^ (6, *N* = 52) = 2.45, *p* = .874.

**Table 4:** Potentially Traumatic Event Exposure Frequency (%) across the Intervention and Training-as-Usual (TAU) Conditions at Baseline (Time 1), Time 2, and Time 3.

	Time 1		Time 2		Time 3	
Freq	TAU	Intervention	TAU	Intervention	TAU	Intervention
0	26.7	20.0	19.6	20.0	9.1	10
1	22.2	13.3	19.6	13.3	22.7	13.3
2	20.0	36.7	10.9	20.0	11.4	13.3
3	17.8	10.0	10.9	6.7	11.4	13.3
4	8.9	3.3	2.2	13.3	9.1	10
5	4.4	3.3	6.5	10.0	6.8	6.7
6	0.0	13.3	6.5	3.3	0	3.3

#### Trauma knowledge

The Condition x Time interaction was significant (*F*[2,182] = 15.13, *p* < .001, η_p_^2^ = .14). The simple main effect of time was significant for the intervention group (*F*[2,182) = 8.75, *p* < .001, η_p_^2^ = .09) but not for the TAU group (*F*[2,182) = 1.39, *p* = .253, η_p_^2^ = .02). Least significant different (LSD) post-hoc contrasts conducted across the simple main effect of time for the intervention group indicated a significant T1 –T2 increase in trauma knowledge (*t*[182] = 3.17, *p* = .002, *d* = .47), which was maintained at T3 (T1 –T3: *t*[182] = 3.80, *p* < .001, *d* = .56). For the adjusted means and standard error of trauma knowledge at Baseline, 6-month follow-up, and 12-month follow-up, see Table 5.

**Table 5:** Adjusted Means and Standard Errors of Outcome Variables at Baseline, 6-month follow up and 12-month follow up for the Intervention and TAU Conditions.

					Intervention Condition			TAU Condition		
Measure	Condition Effect	Time Effect	Condition* Time	η_p_^2^	T1	T2	T3	T1	T2	T3
	*F*(1,182)	*F*(1, 182)	*F*(1,182)		*M (SE)*	*M (SE)*	*M (SE)*	*M (SE)*	*M (SE)*	*M (SE)*
MAPS	4.34	7.54	4.34	0.11	16.57	17.49	17.77	16.62	17.01	16.69
	*p* = .039	*p* = .001	*p* = .039		(0.23)	(0.26)	(0.29)	(0.19)	(0.22)	(0.22)
PCL-C	0.04	2.29	7.90	0.12	20.75	21.73	21.50	23.04	19.75	20.60
	*p* = .838	*p* = .104	*p* = .001		(0.91)	(0.99)	(1.05)	(0.83)	(0.77)	(0.82)
Depression	0.10	1.97	0.45	0.00	3.20	4.17	5.75	3.59	2.93	4.74
	*p* = .747	*p* = .149	*p* = .639		(1.44)	(1.58)	(2.36)	(1.01)	(1.06)	(1.51)
Anxiety	0.47	7.45	0.76	0.16	3.64	2.41	2.21	3.80	3.11	2.90
	*p* = .495	*p* = .001	*p* = .469		(0.85)	(0.58)	(0.55)	(0.68)	(0.63)	(0.58)
Stress	1.18	1.05	0.15	0.006	6.50	5.77	6.11	5.96	4.55	5.32
	*p* = .279	*p* = .353	*p* = .858		(0.87)	(0.87)	(1.03)	(0.64)	(0.83)	(0.90)
SSQ	2.55	1.80	6.09	0.08	4.60	3.97	3.42	4.63	4.47	5.16
	*p* = .112	*p* = .168	*p* = .003		(0.44)	(0.40)	(0.36)	(0.37)	(0.38)	(0.45)
Adaptive Cope	0.02	3.87	0.22	0.04	37.19	33.45	32.67	36.45	33.83	33.66
	*p* = .881	*p* = .023	*p* = .804		(1.84)	(4.40)	(1.87)	(1.48)	(3.30)	(1.58)
Maladaptive Cope	0.24	3.38	0.21	0.05	17.84	16.46	16.67	17.80	15.62	15.88
	*p* = .621	*p* = .036	*p* = .811		(0.88)	(1.83)	(1.11)	(0.68)	(1.27)	(0.80)

#### Social support and satisfaction

The Condition x Time interaction for perceived social support was significant (*F*[2,182] = 5.58, *p* = .004, η_p_^2^ = 06). The simple main effect of time was significant for the control group (*F*[2,182) = 3.39, *p* = .036, η_p_^2^ = .06) but not for the intervention group (*F*[2,182) = 2.55, *p* = .081, η_p_^2^ = .03). LSD (least significant difference) post-hoc contrasts conducted across the simple main effect of time for the control group indicated a significant increase in perceived social support but only at T3 (T1 –T3: *t*[182] = 2.52, *p* = .013, *d* = .58). For the adjusted means and standard error of SSQ at Baseline, 6-month follow up, and 12-month follow-up, see Table 5.

The Condition x Time interaction for social support satisfaction was not significant (*F*[2,182] = 1.52, *p* = .223, η_p_^2^ = .02). The main effects of condition and time can therefore be interpreted independently of one another. The main effect for condition was non-significant (*F*[1,182] = 3.07, *p* = .081, η_p_^2^ = .02), indicating that the two conditions were equivalent in terms of percevied satisfaction with social support at T1, T2, and T3. The main effect for time was also non-significant (*F*[2,182] = 1.42, *p* = .246, η_p_^2^ = .02), indicating that neither group changed significantly across time. These results are inconsistent with the hypotheses.

#### Adaptive coping

The Condition x Time interaction was not significant (*F*[2,182] = 0.22, *p* = .804, η_p_^2^ = .00). The main effects of condition and time can therefore be interpreted independently of one another. The main effect for condition was non-significant (*F*[1,182] = 0.02, *p* = .881, η_p_^2^ = .00), indicating that the two conditions were equivalent in terms of adaptive coping levels at T1, T2, and T3. The main effect for time, however, was significant (*F*[2,182] = 3.87, *p* = .023, η_p_^2^ = .04). LSD post-hoc contrasts conducted across the main effect of time indicated a significant decrease in adaptive coping levels from T1 to T3 (*t*[182] = 2.73, *p* = .007, *d* = .40). For the adjusted means and standard error of adaptive coping at Baseline, 6-month follow-up and 12-month follow-up, see Table 5. These results indicate that both groups significantly decreased at the same rate from T1 to T3, and are therefore inconsistent with our expectations.

#### Maladaptive coping

The Condition x Time interaction was not significant (*F*[2,182] = 0.21, *p* = .811, η_p_^2^ = .00). The main effects of condition and time can therefore be interpreted independently of one another. The main effect for condition was non-significant (*F*[2,182] = 0.25, *p* = .621, η_p_^2^ = .00), indicating that the two conditions were equivalent in terms of maladaptive coping levels at T1, T2, and T3. The main effect for time, however, was significant (*F*[2,182] = 3.38, *p* = .036, η_p_^2^ = .04). LSD post-hoc contrasts conducted across the main effect of time indicated a significant decrease in maladaptive coping levels from T1 to T3 (*t*[182] = 2.48, *p* = .014, *d* = .37). For the adjusted means and standard error of maladaptive coping at Baseline, 6-month follow up and 12-month follow-up, see Table 5. These results indicate that both groups significantly decreased at the same rate from T1 to T3, and are therefore inconsistent with our hypotheses.

#### Psychopathology

*PTSD*. At baseline no participants met the cut-off criteria for PTSD, using the weighted scoring protocol for the PCL-C. At both Time 2 and Time 3, 2 (6.7%) TAU participants appeared to meet the criteria for PTSD, as compared to none (0.0%) of the Intervention participants.

The Condition x Time interaction was significant (*F*[2,182] = 7.90, *p* = .001, η_p_^2^ = .08). The simple main effect of time was significant for the TAU group (*F*[2,182) = 11.02, *p* < .001, η_p_^2^ = .11) but not for the intervention group (*F*[2,182) = 0.72, *p* = .490, η_p_^2^ = .01). LSD (least significant difference) post-hoc contrasts conducted across the simple main effect of time for the TAU group indicated a significant T1 –T2 decrease in PTSD symptoms (*t*[182] = 3.30, *p* < .001, *d* = .49), which was maintained at T3 (T1 –T3: *t*[182] = 2.45, *p* = .002, *d* = .36). For the adjusted means and standard error of PTSD at Baseline, 6-month follow up and 12-month follow-up, see Table 5. These results are inconsistent with our hypotheses.

*Depression*. The Condition x Time interaction was significant (F[2,182] = 4.20, *p* = .017, η_p_^2^ = .04). The simple main effect of time was significant for the intervention group (*F*[2,182) = 3.86, *p* = .023, η_p_^2^ = .00) but not for the TAU group (*F*[2,182) = 1.57, *p* = .210, η_p_^2^ = .02). LSD (least significant difference) post-hoc contrasts conducted across the simple main effect of time for the intervention group indicated a significant T1 –T2 increase in depressive symptoms (*t*[182] = 2.15, *p* = .033, *d* = .50), which was maintained at T3 (T1 –T3: *t*[182] = 2.55, *p* = .012, *d* = .59). For the adjusted means and standard error of depression at Baseline, 6-month follow up and 12-month follow-up, see Table 5. These results are inconsistent with our hypotheses.

*Anxiety*. The Condition x Time interaction was not significant (*F*[2,182] = 0.68, *p* = .507, η_p_^2^ = .007). The main effects of condition and time can therefore be interpreted independently of one another. The main effect for condition was non-significant (*F*[1,182] = 0.00, *p* = .986, η_p_^2^ = .00), indicating that the two conditions were equivalent in terms of anxiety levels at T1, T2, and T3. The main effect for time, however, was significant (*F*[2,182] = 11.52, *p* < .001, η_p_^2^ = .11). LSD post-hoc contrasts conducted across the main effect of time indicated a significant decrease in anxiety for both groups from T1 to T2 (*t*[182] = 4.10, *p* < .001, *d* = .95, and from T1 to T3 (*t*[182] = 4.03, *p* < .001, *d* = .93). For the adjusted means and standard error of anxiety at Baseline, 6-month follow up, and 12-month follow-up, see Table 5. These results are inconsistent with our hypotheses.

*Stress*. The Condition x Time interaction was not significant (*F*[2,182] = 2.21, *p* = .113, η_p_^2^ = .02). The main effects of condition and time can therefore be interpreted independently of one another. The main effect for condition was significant (F[1,182] = 4.35, *p* = .039, η_p_^2^ = .05), indicating that the control group was significantly less stressed than the intervention group at T1, T2, and T3. The main effect for time was also significant (*F*[2,182] = 7.87, *p* = .001, η_p_^2^ = .08). LSD post-hoc contrasts conducted across the main effect of time indicated a significant decrease in stress for both groups from T1 to T2 (t[182] = 3.73, *p* < .001, *d* = 86), and from T1 to T3 (*t*[182] = 2.83, *p* = .005, *d* = .65). For the adjusted means and standard error of stress at Baseline, 6-month follow up, and 12-month follow-up, see Table 5. These results are inconsistent with our hypotheses.

## Discussion

The primary aim of this study was to evaluate the MAPS program in terms of the primary prevention of PTSD in fire and emergency service career recruits. We hypothesised that the intervention group would report fewer symptoms of PTSD and other mental health problems, such as depression, anxiety, and stress, as compared to the TAU group. We found no evidence that MAPS training was effective in the primary prevention of mental health issues, nor did we find any significant impact of MAPS training on social support or coping strategies. Although two TAU recruits (as opposed to none of the intervention recruits) self-reported clinical levels of PTSD symptomatology during their first 12 months as career firefighters, this did not represent a significant difference between the groups.

It was also hypothesised that participation in the MAPS program would influence perceived social support and coping strategies; these hypotheses were not supported. Research published after the design of the current study has suggested that there is no link between social support and treatment seeking in individuals with PTSD and that social support is less important at low levels of PTSD and distress [44,50]. It is plausible that differences in support seeking or perceived social support may be detected over a longer follow-up period or where there is greater variation in symptom severity. Social support is also now recognised as a bi-directional construct [51]; the measure used in the current study measured perceived social support but not provision of social support to others. The sample in the current study commenced recruit school and, for the most part, remained psychologically healthy for the duration of the study. Changes in support seeking and utilisation of coping skills would be expected when under prolonged or intense stress [52] but are not expected to be detected in a sub-clinical and otherwise healthy sample.

Organisational culture, availability of resources, lack of awareness and stigma have been cited as the main barriers to treatment seeking within high risk professions [5,53]. The relationship between social support, support seeking and PTSD development is unclear. The notion that social support can be impacted by targeting awareness and that this will then impact PTSD development is naïve, as the development of PTSD could be influenced by a range of other factors. Avoidance is a key symptom cluster in PTSD [54]; it is unclear from research to-date whether lack of support seeking allows avoidance symptoms to flourish or whether a predisposition for avoidance inhibits support seeking behaviours. If avoidance behaviours are part of inherent personality qualities within an individual then brief support seeking interventions are unlikely to impact the development of the avoidance cluster of PTSD symptoms.

It was also expected that all participants would be exposed to at least one DSM-5 Criterion A [54] PTE during their first 12-months of fire and emergency service work. At 12-month follow up 9.1% TAU and 10.0% Intervention participants reported no lifetime PTE exposures. This is surprising and it does not seem plausible that one could participate in 12-months of fire and emergency work without at least a single exposure to fire, damage to property, injury, mutilation, or death. The measure used (Traumatic Stress Scale) was designed for civilian use. As such, it may be that items were not interpreted as being inclusive of occupational exposures. Past research has adapted the TSS for use in high risk populations, such as law enforcement [55]; this may have been useful in the current study. The impact of cumulative PTE exposure becomes apparent over time and may not be detectable within a 12-month time frame [4].

The initial impression of these results is that the intervention simply did not work in a primary prevention capacity. At the time of writing the intervention, the briefest comparable resilience program comprising multiple skills and strategies with published data was 8-hours in length [42]. Similar resilience programs undergoing evaluation at the time of writing were also typically at least seven hours in length [30]. Eight hours was the program length requested for the MAPS intervention, however due to time and resourcing constraints within the recruit school, 4-hours was granted, making the length of the MAPS intervention less than half that of the nearest comparable study.

An underlying focus of the MAPS program was reducing barriers to treatment seeking. Failure to directly measure help seeking behaviour was a limitation, however given low levels of reported distress and symptomatology help-seeking may not have been necessary during the window of participation. Stress reactions were normalised as part of the normal coping response in a range of ways, including using analogies to physical health. It may be that this normalisation process meant that the intervention recruits were more open in their reporting of distress. Decreased stigma and open reporting of symptoms may mask any potential benefit of a resilience program. Under-reporting of distress or mental health symptoms is typical in high risk and male dominant populations and has been observed in similar studies involving firefighters; the additional inclusion of measures known to be more transparent in fire and emergency populations, such as alcohol use, could have been useful here [56,57].

The significant difference across conditions in trauma knowledge is indicative of some impact of the MAPS program. Unfortunately, alternative possible areas of impact, such as core resilience, post-traumatic growth, feelings of efficacy, agency and competence in managing stress and treatment seeking, were not measured here. Past research indicates that people often experience depreciation as well as growth following PTE exposure [58]. Alcohol use, which has been touted to be a more sensitive measure of stress in populations that are notorious for under-reporting mental health symptoms due to stigma [57], was not measured. High risk, male dominated, populations, such as fire and emergency services, have been documented in the past to under report stress and mental health symptoms. Past emergency services research has found effects on alcohol use and quality of life, with no accompanying changes in self-reported mental health symptoms, indicating that alcohol use may be a better indicator of program effectiveness in some populations [57]. In Australia more men than women report alcohol related issues, while more women than men report mental health issues [59]. The greater range in alcohol use in men and decreased stigma may make this a more sensitive measure for detection of changes in functioning and wellbeing than other self-report measures. This phenomena may have impacted current results. Evaluations of the Australian Defence Force’s BattleSMART resilience training program have shown that the program significantly decreases mental health stigma and barriers to care [60], so it is plausible that the MAPS program also had this effect, resulting in more transparent reporting of mental health symptoms in the intervention group. It is also unclear whether participants applied the MAPS training. Due to resourcing constraints it was not possible to follow-up with participants in more detail about the application of MAPS skills, which could have indicated whether this intervention has real-life practical use.

The exposure time and length of follow-up are also likely to have impacted these results. A longer follow up is needed to fulfil a true longitudinal design and to detect a cumulative toll of fire and emergency work. The floor effect of all participants entering recruit school symptom free following psychological screening and subsequent low base rates of symptomatology prohibited significant findings. Psychological distress among professional firefighters typically peaks after 12.5 years of service and is more common among older firefighters [61]. Similar studies evaluating the impact of resilience training in high risk professions have also reported a floor effect in regards to symptomatology, but due to the null results, outcomes were not published in peer reviewed journals [62]. Additionally, information about appraisal of stressor intensity, a factor impacting mental health outcomes, was not collected [63]

The MAPS training program may have failed to impact mental health symptoms because of statistical, design and power issues, or due to insufficient intervention dosage. However, it is also plausible that the primary prevention of PTSD and other mental health issues via group intervention is simply not effective. Past research has shown that intervening in natural coping responses, as in the case of critical incident debriefing, can be harmful [64]. Recruit training is a time when fire and emergency personnel are acquiring one of their strongest protective factors: their preparedness, competence and professional knowledge [65,66].

### Future Directions and Limitations

While the key hypotheses in the current study were not supported this study is the first randomised control trial investigating the primary prevention of PTSD. Practical barriers around the implementation of this program, including constraints within the recruit school, may inform the design and implementation of similar programs in the future. As long as resourcing is limited and psychological resilience is not prioritised, these barriers to moving towards resilience will remain.

The practical barriers to finding sufficient time within recruit programs to schedule resilience training indicates that recruit school is a time when TFFs are required to learn many other aspects of fire and emergency work. We must consider the possibility that intervening during recruit school could interrupt natural coping responses. The impact of providing resilience training at a later time, when basic training and professional skills have been consolidated and participants are not in the midst of training stress, should be investigated.

In conclusion, the current study did not find evidence for the efficacy of a targeted intervention for the primary prevention of PTSD or other mental health issues in trainee fire fighters at DFES in Western Australia, with all conditions showing a floor effect. The version of the MAPS program trialled in the current study was “watered down” to include only half of the original content due to time constraints within the recruit school; this may account for the unexpected impotency of the program. Trialling resilience or primary prevention programs of increased length (8 hours as opposed to 4 hours) with components that have been shown to be effective in a short-term intervention and including alternate measures of resilience as well as qualitative data collection would assist in further developing knowledge in this area.

## Appendix A

### MAPS Knowledge Questionnaire

Please circle the correct answer.

1. What does MAPS stand for?
  - Momentary Areas of Psychological Stress
  - Moving Around for Physical Strength
  - Mental Agility and Psychological Strength
  - None of the above
2. Which of the following are component of strong MAPS?
  - Take a moment.
  - Assess the situation.
  - Plan your response.
  - Seek Support.
  - All of the above.
3. The physiological or bodily reactions to stress include:
  - Sweating, rapid or shallow breathing, increased heart rate.
  - Anger and frustration
  - Tiredness.
  - All of the above.
4. PTSD is a likely outcome following a ‘traumatic’ event.
  - True
  - False
5. Many people are exposed to trauma.
  - True
  - False
6. How stressful a situation is depends on:
  - Your assessment of the situation.
  - Your assessment of your ability to cope.
  - Whether you feel supported.
  - All of the above.
7. Asking for support shows Psychological weakness.
  - True
  - False
8. The best way to get over something is to avoid thinking about it.
  - True
  - False
9. Self care is selfish.
  - True
  - False
10. Intentionally trying to avoid a thought will increase the frequency and intensity of the thought.
  - True
  - False
11. How often do you need to work on mental fitness to maintain it?
  - Once only.
  - Never.
  - Every 2–4 years
  - Regularly
12. Seeking help and support means:
  - You’re weak
  - You can’t cope
  - You are likely to have stronger mental health
13. Which of the following is a helpful coping strategy?
  - Accepting and making a plan.
  - Watching more TV to relax.
  - Going out drinking
  - Blaming others
14. Which of the following is an unhelpful coping strategy?
  - Seeking support
  - Making an appointment with a counsellor
  - Just trying to think about something else
  - Taking action
15. When people go to counselling or therapy it means:
  - They’re crazy
  - They have personal weakness
  - They have major problems
  - None of the above
16. It is important to know about stress because:
  - It can lease to physical issues
  - It can lead to psychological issues
  - It can interfere with your everyday life and relationships
  - All of the above
17. Which is not an indicator of post-traumatic stress disorder?
  - Flashbacks
  - Insomnia
  - Depression
  - Personal weakness
18. When is it critical to practice self-care?
  - When you have been under stress for more than 6 months
  - Never
  - After a particularly stressful day or when you are starting to notice symptoms of stress
  - When you are hung-over
19. The best way to relax after a stressful day is to:
  - Drink alcohol
  - Get lost in video games
  - Ignore everyone
  - None of the above
20. Physical fitness has nothing to do with mental wellbeing.
  - True
  - False

## Supporting Information

S1 Data MAPS data. (SAV)

S1 Document Ethics Form A. (PDF)

S2 Document Ethics Form B. (PDF)

S3 Document CONSORT Checklist. (DOC)
