# Supplementary material for: The Primary Prevention of PTSD in Firefighters: Preliminary Results of an RCT with 12-Month Follow-Up
Source: PLoS One. 2016 Jul 6;11(7):e0155873. doi: 10.1371/journal.pone.0155873 (PMC4934826; doi:10.1371/journal.pone.0155873)
Supplement: S1 Document — (PDF) [file pone.0155873.s002.pdf]

HUMAN RESEARCH ETHICS COMMITTEE

Application for Ethical Approval of a Research Project Involving Humans (Form A)

INFORMATION FOR APPLICANTS

1. Ensure that you are using the most current version of the form.

2. Hand written applications are not accepted.

3. Please ensure that you are aware of the deadlines dates http://research.curtin.edu.au/guides/human.cfm.

4. Applications will only be processed when all copies are received (both hard and electronic).

LODGING YOUR APPLICATION

You must submit:

1. Three (3) signed hardcopies: (1) original and (2) double sided photocopies, of the application and all supporting documentation to:

**In Person**	**Posting**
The Secretary<br>Human Research Ethics Committee<br>Office of Research and Development<br>Curtin University of Technology<br>Building 100, Level 1 West<br>Bentley WA 6845	The Secretary<br>Human Research Ethics Committee<br>Office of Research and Development<br>Curtin University of Technology<br>GPO Box U1987<br>Bentley WA 6845

2. A full electronic version of the application and all supporting documentation submitted to ethicsapplications@curtin.edu.au. Please ensure all attachments are clearly labelled and in pdf format (if possible).

FILLING IN YOUR APPLICATION

- This form is set up as a series of tables and check boxes ☐.

- The table will enlarge to the size you require when you type and by pressing the Enter key.

- Double click on the left mouse button ☒ and a "check box form fields" box will appear. Choose CHECKED and OK.

- If you want to uncheck it, double click on the left mouse button ☐ and a check box form fields box will appear. Choose NOT CHECKED and then click on OK.

- Note: if there are more than 2 co-investigators please use the additional page and attached to your application

  http://research.curtin.edu.au/guides/forms/ethicsforms.cfm#HumanResearchEthics (2nd indented dot point)

Do not submit this page with your application.

FORM A
Application for Ethical Approval of Research Involving Humans

Office use only HR__________

SECTION 1	
**Project title**	Developing a theory driven and evidence based targeted intervention for the primary prevention of PTSD.
**Project summary**<br>*A plain English description of the project and its projected outcomes in no more than 100 words*	The current 'best practice' approach to trauma is to screen for pathology and provide treatment when required (Cloitre, 2009; Cornum, Matthews & Seligman, 2011). Only in recent months have studies reflecting resilience training programs with adults appeared (Burton, Pakenham and Brown, 2010; Cornum et al, 2011). Some professions, by their nature, will be exposed to trauma. Policing is widely recognised as a stressful occupation, with police officers often exposed to potentially traumatic situations. Police officers have abnormally high rates of stress related negative outcomes such as alcoholism, physical health problems and divorce (Pole et al 2006).<br>This research project aims to explore the possibility of teaching resilience to adults in high-trauma professions by developing and evaluating an evidence-based, targeted intervention for the primary prevention of PTSD in police officers.
**Principal investigator**	
(Only current Curtin University of Technology staff can be named as the Principal Investigator. This is the person with whom the ultimate responsibility for the project lies. If it is a student project, provide the student's details under co-investigator)	
**Title and Name**	Assoc. Prof Clare Rees
**Staff ID**	167815e
**Department/School**	School of Psychology and Speech Pathology
**Email**	c.rees@curtin.edu.au
**Phone**	3442
**Describe what this researcher will do in the context of this project**	Supervise the PhD Candidate
**Include a brief summary of relevant experience for this project**	Experienced Clinical Psychologist. Experienced research supervisor.
**Co investigator**	
**Title and Name**	Ms. Petra Skeffington
**Staff ID/ Student ID**	15481687
**Department/School**	School of Psychology and Speech Pathology
**Email**	petra.skeffington@postgrad.curtin.edu.au
**Phone**	0402 312 619
**Describe what this researcher will do in the context of this project**	Conduct research: design project, gather and analyse data, write-up.

**Include a brief summary of relevant experience for this project**	Independent research for previous Honours and Masters dissertations; work experience as a research assistant.

Co investigator	
Title and Name	Dr Robert Kane
Staff ID/ Student ID	175232E
Department/School	School of Psychology and Speech Pathology
Email	r.t.kane@curtin.edu.au
Phone	0405292735
Describe what this researcher will do in the context of this project	Dr Kane will provide advice on methodology and data analysis.
Include a brief summary of relevant experience for this project	Experienced statistician with 35 years experience supervising post-graduate students.

Project Supervisor<br>(if applicable)	
Title and Name	
Staff ID	
Department/School	
Email	
Phone	
Describe what this researcher will do in the context of this project	
Include a brief summary of relevant experience for this project	
Project or application type	☒ STUDENT please specify<br>(i) Doctoral (eg, PhD) ☒ (iii) Master's by Coursework ☐<br>(ii) Master's by Research ☐ (v) Honours ☐<br>(iv) Undergraduate ☐<br><br>☐ STAFF<br>☐ EXTERNAL

NB: If there is more than two co-investigators please use the 'Additional Co-Investigator' page and attach to your application http://research.curtin.edu.au/local/docs/ethics/HREC_FORM%20A%20extra%20co-investigators.docx

Research involving humans
Before conducting research that involves humans as participants, you must receive approval from the Human Research Ethics Committee. If you do not have an approval number, do not start your research. In conducting such research, all researchers must also read and abide by the Australian Code for the Responsible Conduct of Research.
Research involving humans should always comply with current ethical standards. In Australia, the ethical standards for such research are set by the National Health and Medical Research Council (*the NHMRC) National Statement on Ethical Conduct in Human Research* and those proposing to carry out research should be familiar with publications of the NHMRC. See http://www.nhmrc.gov.au/publications/synopses/e72syn.htm

The aim of ethical review of human research is to ensure that participants in research are not put at risk of harm, are not disadvantaged and are made aware that they may withdraw without prejudice.

Broadly, the process of ethical review concentrates on three main areas:

A Gathering informed consent to participate in research projects

B Protection of privacy and confidentiality of records

C Risk of harm to subjects or to groups in the community

In the following section you are asked to answer a number of questions under each of these three headings in order to identify any ethical considerations that may arise from your proposed research. Following this set of questions there is a further check list relating to types of research that have previously been identified as likely to raise ethical questions. In the the second check list each of the types of research is cross referenced to a chapter of the NHMRC guidelines for you to read.

The following checklist is designed to alert you to the major types of ethical issues in your research. If you answer Yes to any of these questions, be sure to explain and clarify the issue elsewhere in the document.

Ethical Issues Checklist

A: Informed consent.
Research subjects must be able to give consent to their participation in research in such a way that ensures that they are fully informed of relevant aspects of the research and that they are confident to give consent for the research to be undertaken. Researchers should ensure that individuals are not directly or indirectly pressured or coerced into participation through unequal power relationships or payments or inducements. The use of deception in any form in a research protocol has the potential to prevent the subject from giving consent that is truly well-informed.

Does your research involve: (please tick)

1. Processes that potentially exclude and/or disadvantage a person or group, such as the collection of information which may expose the person/group to discrimination or misrepresentation?	YES ☐ NO ☒
2. Collection or disclosure of personal information by a Commonwealth, State or Territory agency that might involve a breach of an Information Privacy Principle (as defined by the Commonwealth Privacy Act 1988 and the Australian Standard)?	YES ☐ NO ☒
3. Collection or disclosure of personal information by a private sector organisation [that might involve a breach of a National Privacy Principle (as defined by the Commonwealth Privacy Act 1988)]?	YES ☐ NO ☒
4. Payments or inducements, other than reasonable recompense, to participants for their participation?	YES ☐ NO ☒
5. Deception of the participants including concealment and covert observation?	YES ☐ NO ☒
6. Disclosure of the response outside the research which could place the participants at risk of criminal prosecution or civil liability or be damaging to their financial standing, employability, professional or personal relationships?	YES ☐ NO ☒
7. Any form of passive consent?	YES ☐ NO ☒

B: Risks to privacy and confidentiality.

The privacy of individuals and the confidentiality of data are both vital. The research must take special care to protect the privacy and confidentiality of subjects and the data obtained from them.

Does your research involve:

8. The participation of minors (under 18 years), other than in the observation of normal school activity?	YES ☐ NO ☒
9. Participants who are in a dependent situation, such as students or residents of an institution (such as a hospital, nursing home or prison or patients highly dependent on medical care), other than those who are being observed in their normal environment where such observation is considered innocuous?	YES ☐ NO ☒
10. Participants who may be unable to give or are incapable of giving informed consent?	YES ☐ NO ☒
11. The participation of Aboriginal or Torres Strait Islanders, or other peoples from identifiable cultural, ethnic or minority groups?	YES ☐ NO ☒
12. a) Acquisition of data about organisations or individuals through any form of database at any stage of the research?	YES ☐ NO ☒
b) Organisations or individuals who are directly or indirectly identifiable by the researcher within the database?	YES ☐ NO ☒
13. Use of questionnaires or interviews, which may be, linked either directly (eg through recording of names) or indirectly (eg through a cross-linked code) to the individual/ participant/researcher at any stage of the research, including the obtaining of data?	YES ☒ NO ☐
14. Use of questionnaires, interviews, or procedures, irrespective of the recording of the individual's identity, which might reasonably be expected to cause discomfort, embarrassment, or psychological or spiritual harm to the participants?	YES ☐ NO ☒

C: Is there a risk of harm to subjects or groups in the community?

Individuals may be put at risk through the use of new and untried procedures, invasive procedures, the administration of drugs, or the use of procedures likely to cause pain or suffering. Individuals and groups in the community may be also be harmed through damage to their cultural security or through processes which might expose them to discrimination or misrepresentation.

Does your research involve:

15. Any novel procedure in the therapy or management of patients in a clinical setting?	YES ☐ NO ☒
16. Any form of physically invasive procedure such as blood collection, exercise regimens or physical examination, and which is not part of clinical management?	YES ☐ NO ☒
17. Any form of physically invasive procedure on volunteer participants such as body fluid collection (eg blood, urine, semen), exercise regimens or physical examination?	YES ☐ NO ☒
18. The administration of any form of drug, medicine (other than in the course of standard medical procedure) or placebo?	YES ☐ NO ☒
19. Physical pain, beyond mild discomfort?	YES ☐ NO ☒
20. Obtaining and storage of blood, body fluid or tissue samples from the participants?	YES ☐ NO ☒

21. Any other ethical issue of the study which has not been addressed in this Checklist?	YES ☐ NO ☒

FINAL CHECKLIST

As a final check, please respond to the following list of research areas that commonly raise ethical concerns. Research involving any of the categories listed below is subject to compliance with the provisions of the NH&MRC *National Statement on Ethical Conduct in Human Research*. If you answer Yes, or Probably, please ensure that you have explained and clarified each item elsewhere in the document and that you have both read the relevant chapter of the National Statement (http://www.nhmrc.gov.au/publications/synopses/e72syn.htm) and addressed the specific considerations therein.

Does this proposal involve: -

Please tick answers to ALL questions

Does this proposal involve: -	YES	POSSIBLY	NO
1. minors i.e., under the age of 18 (chapter 4.2)?	☐	☐	☒
2. persons with an intellectual or mental impairment (chapter 4.5)?	☐	☐	☒
3. persons highly dependent on medical care (chapter 4.4)?	☐	☐	☒
4. persons in dependent or unequal relationships (chapter 4.3)?	☐	☒	☐
5. collectivities (such as other specified racial groups) (chapter 4.8)?*	☐	☐	☒
6. separate identification of, or focus on, Aboriginal and Torres Strait Islander peoples (chapter 4.7)?*	☐	☐	☒
7. ionising radiation (X-rays, fluoroscopy or radioisotopes) (chapter 3)?**	☐	☐	☒
8. assisted reproduction technology (chapter 3)?	☐	☐	☒
9. clinical trials (chapter 3)?	☐	☐	☒
10. innovative therapy or intervention (chapter 3)?	☐	☐	☒
11. epidemiological research (chapter 3)?	☐	☐	☒
12. use of human tissue samples (chapter 3.4)?	☐	☐	☒
13. human genetic research (chapter 3.5)?	☐	☐	☒
14. any perceived, possible or actual conflicts of interest#	☐	☐	☒
15. a researcher or organisation with which any of the research team has a commercial relationship#	☐	☐	☒
16. the use of Curtin University students as participants#	☐	☐	☒
17. the use of any participants with which the researcher has a relationship such as teacher-student; line manager-staff; employer-employee#	☐	☐	☒

**If you answered yes to questions 5 or 6, have you consulted the Guidelines for Ethical Conduct in Aboriginal and Torres Strait Islander Health Research. (http://www.nhmrc.gov.au/publications/synopses/e52syn.htm)* *This provides good guidance on dealing with cultural groups and their sensitivities* *# If you answered yes to any of questions 14,15,16 or 17 please provide a clear written explanation as an appendix to this application.* ***For research involving ionising radiation, microwaves, lasers or ultraviolet light, researchers must submit a separate application to the Radiation Safety Officer, for consideration of approval by the Radiation Safety Committee. research cannot commence without such approval*	YES ☐ NO ☒

Please indicate the National Statement chapters you have consulted (for Questions 1-13)	Chapters 3, 4

For each item to which you have ticked **Yes** or **Possibly**, please state briefly how your research complies with the relevant section of the National Statement. (For Question 1-13)

Participants in this study will possibly be in an dependent or unequal relationship to the research. This research project is designed to integrate into the profesisonal training program for WA Police Recruits. Recruits will be informed of this and will have the option to train with another school, if they do not wish to participate. Training will be delivered by a member of the WA Police Psychology Unit rather than by the researcher in order to avoid dual relationship issues.

(i) What is the estimated completion date of the project?

December 2014

(ii) Has an application been made for a research grant for this project? If YES, please state the name of the granting body and the status of the application.

YES ☐ **NO** ☒

(iii) Please state the sources of all funds to be used for this project.

(iv) Will you or any of the researchers receive any sort of remuneration or reward from non-University Sources for work done in this research.
If YES, please provide details.

YES ☐ NO ☒

(v) Has this project been approved by the Curtin Human Research Ethics Committee previously? If YES, please quote the approval number.

YES ☐ **NO** ☒

HR_________________

(vi) Is this project part of a larger study? If YES, please provide details.

YES ☐ **NO** ☒

(vii) Is this project part of a multi-centre research project? If YES, please provide details of the other centres and the approval status of the study at each centre.

YES ☐ **NO** ☒

(viii) Has this project been submitted or is it likely to be submitted to any other ethics committee? If YES, please supply details including approval dates and approval number. *Attach a copy of all relevant approvals*.

YES ☐ **NO** ☒

(ix) Provide a brief description of the participants/collectivities involved.

Participants will be approximately 120 recruits in the WA Police.

(x) How will participants be recruited? *Researchers who would like permission to have access to the personal details of staff or students of Curtin for the purposes of directly inviting them to participate in a research study (e.g. contact details) will require written approval from the Human Research Ethics Committee.*

Participants will be involved in this research as part of their professional training in the WA Police. Four schools (or cohorts) from the WA Police Academy will be randomised into Intervention or Training-As-Usual (TAU) conditions. Recruits in the intervention condition, will experience the intervention as a component of their 30-week professional training at the WA Police Academy. Participants in the TAU condition will experience the same 30-week training program, without the 8-hour intervention. Recruits will have an option to change to a non-research related school if they do not wish to participate.

(xi) Will personal (identified) data be obtained from a Commonwealth Agency? If **YES**, **please specify**, e.g. Department of Foreign Affairs.
(see Section 1.1 of the *Guidelines under Section 95 of the Privacy Act 1988*, "The use of the Guidelines" *http://www.nhmrc.gov.au/publications/synopses/e26syn.htm*)

YES ☐ **NO** ☒

(xii) Will **health** information data be collected from an **organisation in the private sector** (i.e. not from a Commonwealth or State government agency)? e.g. use of patient information from a private hospital. If YES, please specify the organisation and type of data, and answer questions (a) – (d) below.
(see *Guidelines under Section 95A of the Privacy Act 1988*, page 5; pages 11-17 and pages 35 – 44 the 'National Privacy Principles (NPPs)') *http://www.nhmrc.gov.au/publications/synopses/e26syn.htm*

YES ☐ **NO** ☒
(if No, go to Section 2 "Protocol" below)

Organisation from which health information data will be collected:

The number or records involved:

Description of data to be collected:

xii(a) Does the data include information that identifies the individual(s) involved? **YES** ☐ **NO** ☐
if yes, go to (b)

xii(b) If you are using data that may identify individuals, could the research be conducted using de-identified information? **YES** ☐ **NO** ☐
if no, go to (c)

x(c) If you can identify an individual, is the use of the data or the disclosure of identity something that the individual could reasonably expect to happen? **YES** ☐ **NO** ☐
if no, go to (d)

x(d) Is it proposed to undertake the research, with the consent of the individual(s) involved? **YES** ☐ **NO** ☐
If no, then Section 95A Guidelines will be applied.

End of Section 1

SECTION 2 – PROTOCOL

The main concern of the Human Research Ethics Committee in evaluating proposals is to establish conformity with the NHMRC *National Statement on Ethical Conduct in Human Research.* Researchers must comply with the provisions of the National Statement. Section 1 *Values and Principles of Ethical Conduct* and Section 5 *Processes of research governance and ethical review* are essential reading for all applicants prior to completion of the following questions. **All questions must be answered**. Applicants are required to provide a brief summary **in the spaces provided**. This will assist in expediting the review process. Non-compliance with this request will result in the application being returned to the applicant.

1. Briefly describe (in point form and in less than 100 words) your proposed procedure including: recruitment of subjects, experimental design and/or procedure and analysis of data. *An essential condition of the ethical acceptability of research is the determination that the scientific quality of a proposal is such that the objectives of the proposal can reasonably be expected to be achieved.*

Study 1
- Approximately 500 police officers will be sent an electronic invitation to complete some measures regarding professional exposure to Potentially Traumatic Events (PTEs), symptoms of PTSD, depression, anxiety, stress and alcohol use.
-This will provide a current picture of exposure to PTEs and accompanying symptomatology in the WA Police.
-Analysis by descriptive statistics. Correlations can also be used to verify appropriateness of control variables in study 2.

Study 2
- An 8 hour Psychological Strength training program will be delivered by the WA Police Psychology unit to approximately 40 WA Police recruits. An Additional 40 recruits will complete training-as-usual (TAU).
- A Pre-post-follow up control group design will be used, with two follow up data collection points at 6 months and 12 months post intervention.
- Analysis by Multi-level linear regression.

2. Provide sufficient procedural/experimental detail to enable the Committee to judge whether any <u>risks</u> to which the participants may be exposed are warranted by the possible benefits/outcomes of the study. How will the researcher deal with situations in which participants are identified to be at risk?
Your answer must demonstrate that the welfare, rights, beliefs, perceptions, customs and cultural heritage of the participants are honoured; the risks of harm or discomfort to participants is minimised; and that respect for the dignity and well being of the participants takes precedence over the expected benefits. Consult the Guidelines for Ethical Conduct in Aboriginal and Torres Strait Islander Health Research for guidance on how best to honour welfare, rights, beliefs, perceptions, customs and cultural heritage of the participants. http://www.nhmrc.gov.au/publications/synopses/e52syn.htm

Study 1
An electronic survey invitation will be extended to a random sample of approximately 500 police officers. The invitation will outline information regarding the survey, such as the kinds of questions asked and approximate completion time. Participation will be anonymous and participants can prvide consent electronically before commencing the survey. Participants will be asked a small number of demographic and consultation questions (to assess perceived need or desire for a psychological strenth training program), PTSD symptoms will be measured using the PTSD Checklist, PTE exposure will be measured using the Traumatic Stress Schedule, alcohol use will be measured using the Alcohol Use Disorders Identification Test and depression, anxiety and stress will be measured using the Depression, Anxiety and Stress Scale.

Study 2
Members of the WA Police Psychology Unit will be trained to deliver the intervention, which will be included as part of the professional police training. The intervention will be run over 8 hours. Participants will be asked to complete the above measures, as well as a trauma knowledge measure (to assess whether psycho-educational aspects of the program ae effective), the Social Support Questionnaire- Short Form (to measure perceived scial support) and the Brief COPE (to measure coping style) at pre-intervention, post-intervention, 6 month follow up and 12 month follow up. At pre-intervention only participants will also be asked about treatment credibility and expectations. At post-intervention only participants will be asked about their satisfaction with the program.

3. Describe how participants will <u>consent</u> to participate in the study, and how they are informed of their rights.
Attach copies of the Participant Information Sheet and Consent Form intended for use. Approval cannot be granted until these documents have been submitted. *Your answer should demonstrate that the provisions of Section 1 of the National Statement have been satisfied*.

Study 1<br>Participation will be anonymous and voluntary and all relevant information will be outlined prior to commencement. No identifying information will be collected. The attached information sheet and consent forms are examples to illustrate the content of each form. As this phase of the research will be completed electronically, consent will be provided by the click of an 'accept' button prior to commencing the survey, rather than by filling out a hard copy form.<br><br>Study 2<br>Participation will be voluntary and all participants will be informed of the procedure prior to commencement. Recruits who do not wish to particiate will be able to transfer to another school to complete training as usual.

4. Describe the extent to which issues of privacy are to be addressed in relation to the collection of data from individuals or groups, and the extent to which the collection intrudes upon the personal affairs of the individual or group.

Refer to the National Privacy Principles (see the NHMRC *Guidelines under Section 95A of the Privacy Act 1988 http://www.nhmrc.gov.au/publications/synopses/e26syn.htm)*

Your response should specifically address:-

a. Justification if identified or potentially identifiable information is to be used rather than de-identified information
b. Justification if consent is not being sought to use personal information.
c. The specific uses to which the personal information used during the study will be applied.
d. The proposed method of publication of results of the research

Study 2 only: The identity of participants will be known to the researcher.<br>Given the longitudinal design of Study 2, the researcher will be able to identify participants in order to match data across time periods. The researcher will assign an identifier to each participant and all information will be deidentified upon receipt

5. Provide details of the storage and security arrangements for personal information that will be collected within the study to ensure confidentiality.

Where personal information about research participants or a collectivity is collected, stored, accessed, used, or disposed of, a researcher must strive to ensure that the privacy, confidentiality and cultural sensitivities of the participants and/or the collectivity are to be fulfilled. Refer to the Joint NHMRC/AVCC Statement and Guidelines on Research Practice, Section 2 'Data Storage and Retention' (http://www.nhmrc.gov.au/funding/policy/code.htm).

Your response should address: -

a. The estimated time of retention of the personal information
b. The identity of the custodian(s) of the personal information used during the research
c. Security standards to be applied to the personal information
d. List of personnel with access to the personal information
e. Standards that will be applied to protect personal information disclosed by a Commonwealth agency or private sector organisation (if applicable)
f. The media or forms of the data that are to be stored. For example, electronic data on floppy disc, hard copies, cassette tapes, field samples, photographs, video tape, etc.

All data will be stored securely in a locked filing cabinet within assoc. Professor Clare Rees' office at the School of Psychology and Speech Pathology at Curtin University. Access will be available only to Petra Skeffington, Associate Professor Clare Rees and Dr Robert Kane. The data will be stored for a period of five years after the completion of the project, after which it will be destroyed according to Curtin University Functional Records Disposal Authority protocol.

6. Provide a description of any survey instruments/questionnaires intended for use in the study, including questions/material intended for interviews/workshops and semi-structured interviews. All such material must be submitted for approval. If the instrument has not been designed at the time of application, then a brief description of the anticipated nature of the questions must be provided. Instruments that are widely recognised as being standard in the field should be identified as such, or be available for viewing upon request.

Final approval will be dependent on the satisfactory submission of all instruments.

Demographics and Consultation. A short demographic questionnaire has been designed to gather information relating to age, gender, education, length of service and marital status. This questionnaire includes a consultation component, to ascertain interest in and perceived need or desire for a psychological strength training program, as well as perceptions of current support.

PTSD Symptoms. The PTSD Checklist - Civilian Version (PCL-C;Blanchard, Jones-Alexander, Buckley, & Forneris, 1996) will be used to assess PTSD symptom presence and severity. The PCL-C is a 17-item inventory that assesses the specific symptoms of PTSD. The respondent is asked to rate how much the problem described in each statement has bothered him or her over the past month on a 5-point scale ranging from 1 (not at all) to 5 (extremely). The PCL-C takes 5 to 10 minutes to administer; a total score is an indicator of PTSD symptom severity (Orsillo, 2001).

PTE exposure. The Traumatic Stress Schedule (Norris & Hamblen, 2004) is a 9-item instrument developed to examine the frequency of nine types of traumatic events (robbery, physical assault, sexual assault, tragic death, motor vehicle crash, combat, fire and natural disaster) and has been shown to have good stability, test-retest validity and symptom reliability (Norris & Hamblen, 2004). Based on research by Stephens and Miller (1998), five other items in the same format but relating specifically to police duties have been added. These items were based on the circumstances listed by the NZ Police trauma policy for which mandatory debriefing is required: deliberate killing by police officers, death of a known police officer, accidental death or injury of a member of the public by a police officer, work with victims of horrific homicides, attendances at severe accidents and disaster victim identification work (Stephens & Miller, 1998).

Alcohol Use. The Alcohol Use Disorders Identification Test (AUDIT) is a simple 10-question test developed by the World Health Organisation to determine if a person’s alcohol consumption may be harmful (Babor, Higgins-Biddle, Saunders, & Monteiro, 2001).

Other symptoms. A short version of the Depression, Anxiety and Stress Scale (Lovibond & Lovibond, 1995) will be administered to measure any current symptoms of depression, anxiety and stress. The DASS is a set of three self-report scales designed to measure the negative emotional states of depression, anxiety and stress. Each of the three DASS scales contains 14 items, divided into subscales of 2-5 items with similar content.Subjects are asked to use 4-point severity/frequency scales to rate the extent to which they have experienced each state over the past week.

Trauma knowledge. A short (approx. 20 multiple choice item) assessment of trauma knowledge will be administered to assess whether the psycho-educational component of the intervention effectively improves knowledge of trauma. Items on this assessment will be directly related to information provided during the psycho-educational phase of the intervention, as such, this measure will not be developed until finalisation of the intervention.

Perceived Social Support. The Social Support Questionnaire- Short Form (Sarason, Sarason, Shearin, & Pierce, 1987) is used to quantify the availability and satisfaction with social support that an individual has. It is a 27 -item self-administered scale. Each item involves two parts: respondents are asked to list the individuals who are available to them for help in specific situational circumstances, and how satisfied they are with the support available. Each situational circumstance allows a participant to list up to nine individuals. A satisfaction rating for each situational circumstance is the same regardless of the situation given. A 6- point rating scale (from “very satisfied” to “very dissatisfied”) is used to rate the individual’s satisfaction with his or her support available.

Coping strategies. The Brief Coping Orientations to Problems Experienced (Brief COPE) scale is a 28-item scale used to measure a broad range of cognitive and behavioural coping strategies that individuals typically use in stressful situations (Carver, 1997). It includes 14 subscales: active coping, planning, positive reframing, acceptance, humour, religion, emotional support, instrumental support, self-distraction, denial, venting, substance use, behavioural disengagement and self-blame.

Treatment credibility/ Expectations (Pre-intervention only). Items on this assessment will be directly related to information provided during the psycho-educational phase of the intervention; as such, this measure will not be developed until finalisation of the intervention.

Satisfaction (Post-intervention only). Items on this assessment will be directly related to information provided during the psycho-educational phase of the intervention; as such, this measure will not be developed until finalisation of the intervention.

7. Attach a detailed description of the project using the headings below.

- Aims/objectives of the study
- Background
- Significance/Justification of the study
- Methods (including - data to be collected and source of data; target population; study period; participant recruitment procedures, instruments)
- References

Do not attach copies of grant applications
Recommended length = maximum 10 pages (one and a half line spacing), excluding references. Research students may alternatively attach a copy of their candidacy research proposal. Pages must be numbered. Applicants are reminded to use non-specialist language.

SIGNATURES (Required)

Principal Investigator		Date	
Co-investigator		Date	
Co-investigator		Date	1. 8. 2011
Project Supervisor (if applicable)		Date	

In signing this as Head of School (or equivalent) I confirm that the researchers have the resources and the capacity to undertake the research outlined in this application.

Head of School (PRINT NAME)			
Head of School		Date	

RESEARCH METHOD (WHERE CO-INVESTIGATOR IS A HIGHER DEGREE BY RESEARCH STUDENT)

☐ Application for Candidacy was approved by the Faculty Graduate Studies Committee at the meeting held on ____________ *(dd/mm/yy)*

or

☐ Application for Candidacy has been submitted to the Faculty Graduate Studies Committee for consideration at the meeting scheduled for ____________ *(dd/mm/yy)*

or

☐ Application for Candidacy has not been submitted to the Faculty Graduate Studies Committee but will be submitted for consideration at the meeting scheduled for ____________ *(dd/mm/yy)*
