# Supplementary material for: The Primary Prevention of PTSD in Firefighters: Preliminary Results of an RCT with 12-Month Follow-Up
Source: PLoS One. 2016 Jul 6;11(7):e0155873. doi: 10.1371/journal.pone.0155873 (PMC4934826; doi:10.1371/journal.pone.0155873)
Supplement: S2 Document — (PDF) [file pone.0155873.s003.pdf]

Curtin University

Curtin HREC Form B

PROGRESS REPORT
or APPLICATION FOR RENEWAL

The Form B is to be completed and returned to ***the Secretary, Human Research Ethics Committee, c/- Office of Research & Development.***

If any of the points below apply prior to the expiry date, this form should be submitted to the Committee at that time. An application for renewal may be made with a **Form B three years** running, after which a 'new' application form, providing comprehensive details, must be submitted.

**Approval Number:**	HR113/2011	**Expiry Date** 4/10/2012
**PROJECT TITLE:**	**Developing a theory driven and evidence based targeted intervention for the primary prevention of PTSD**	

1A	Has this project been completed?	YES ☐	NO ☒
1B	OR<br>Do you wish to apply for a renewal of the project?	YES ☒	NO ☐
If YES please state the expected completion date.		1/1/2015	
If NO please state why, eg abandoned/withdrawn/no funding etc.			
2	Has this project been modified or changed in any manner that varies from the approved proposal?	YES ☒	NO ☐
If yes, please provide details______<br>*(Attach additional comments on a separate sheet of paper if necessary)*<br>This project will now be completed in alliance with FESA (rather than the WA Police). The ethical issues have not changed. Written support from FESA for this research alliance is attached.			
3	Have any ethically related issues emerged in regard to this project since you received Ethics' Committee approval? (e.g. breach of confidentiality, harm caused, inadequate consent or disputes on these).	YES ☐	NO ☒
If yes, please provide details<br>*(Attach additional comments on a separate sheet of paper if necessary)*			
4	Have any ethically related issues in regard to this project been brought to your attention by others? (i.e. study respondents, organisations that have given consent, colleagues, the general community etc).	YES ☐	NO ☒
If yes, please provide details ______<br>*(Attach additional comments on a separate sheet of paper if necessary)*			

Investigator:	Petra Skeffington	Signature:	
Co-Investigator:	Dr. R. Kane	Signature:	
School/Department:	School of Psychology & Speech Pathology		
Head of Enrolling Area:		Signature:	
Date:	10/8/2012		

Office Use Only
APPROVED: ___________________________________ DATE: _______/______/_______
Executive Officer
